# Diagnostic value of FNAC combined with BRAF^V600E^ mutation detection in Hashimoto’s thyroiditis complicated with papillary thyroid carcinoma

**DOI:** 10.3389/fendo.2024.1366724

**Published:** 2024-05-16

**Authors:** Jingyao Fu, Xiangdang Yin, Xiaochun Wang, Siqi Xiao, Xianji Wu, Chengcheng Duan, Wenxi Yu, Guang Zhang

**Affiliations:** ^1^ Department of Thyroid Surgery, China-Japan Union Hospital of Jilin University, Changchun, Jilin, China; ^2^ Department of Oral-Maxillofacial-Thyroid Oncosurgery, Jilin Cancer Hospital, Changchun, Jilin, China

**Keywords:** papillary thyroid carcinoma, Hashimoto’s thyroiditis, BRAFV600E mutation, fine needle aspiration cytology, diagnosis

## Abstract

**Background:**

This study aimed to analyze the effect of preoperative fine needle aspiration cytology (FNAC) combined with BRAF^V600E^ mutation detection as compared to that of fine needle aspiration cytology alone on the diagnostic performance of papillary thyroid carcinoma (PTC) combined with Hashimoto’s thyroiditis (HT).

**Method:**

Patients with thyroid nodules in Hashimoto’s thyroiditis, who underwent fine-needle aspiration cytology examination and BRAF^V600E^ mutation detection in the puncture eluate at the outpatient clinic, were selected. Finally, 122 patients received surgical treatment and were included in the study. We used postoperative pathological results as the gold standard. Accordingly, we compared the sensitivity, specificity and accuracy of preoperative FNAC alone and FNAC combined with BRAF^V600E^ mutation detection in for the diagnosis of PTC combined with HT.

**Results:**

For PTC patients with HT, the sensitivity of FNAC diagnosis was 93.69%, the specificity was 90.90% and the accuracy was 93.44%. However, the sensitivity, specificity and accuracy of FNAC combined with BRAF^V600E^ mutation detection were 97.30%, 90.90% and 96.72%, respectively. Therefore, combined detection can improve the sensitivity and accuracy of diagnosis (p<0.05).

**Conclusion:**

FNAC combined with eluent BRAF^V600E^ mutation detection can improve the sensitivity and accuracy of diagnosis of PTC in the background of HT.

## Background

Papillary thyroid carcinoma (PTC) is the most common pathological subtype of all thyroid cancers. PTC accounts for about 80%-85% (1) cases. However, it has a better prognosis than other thyroid malignancies. At present, fine-needle aspiration cytology (FNAC) is the most reliable and cost-effective diagnostic tool to determine benign and malignant thyroid nodules. Notably, FNAC can distinguish 70%-80% of thyroid nodules as benign or malignant (2). The sensitivity and specificity of FNAC are 65%-99% and 72%-100%, respectively (3).

Hashimoto’s thyroiditis (HT) is the most common autoimmune inflammation of the thyroid. The incidence of HT combined with PTC has been increasing in recent years and the relationship between PTC and HT warrants further research. The presence of HT significantly reduced the sensitivity and specificity of FNAC in diagnosing benign and malignant thyroid nodules, and increased the probability of false-negative detection of FNAC and indeterminate cytology results (4). The standard of diagnosis and treatment of thyroid carcinoma in China (5) indicates that for benign and malignant nodules that cannot be determined by FNAC, preoperative detection of relevant molecular markers such as BRAF^V600E^ mutation and RAS mutation can help in improving the accuracy of diagnosis. Previously, several molecular markers have been confirmed to be associated with PTC combined with HT, such as BRAF^V600E^ mutation and RET/PTC rearrangement, among others (6, 7).

BRAF^V600E^ mutation is one of the most common mutations in papillary thyroid carcinoma and it has an incidence of about 38%-90% (8–10). Further, BRAF^V600E^ mutation leads to the abnormal activation of the MAPK pathway, which promotes the occurrence and development of PTC. Multiple studies have shown that BRAF^V600E^ mutations are associated with the invasiveness of PTC (11). However, the association of HT and BRAF^V600E^ mutations with the clinical and pathological features of PTC remains unclear. Our study aimed to examine whether FNAC combined with BRAF^V600E^ mutation detection could improve the diagnostic accuracy of PTC in the background of HT.

## Method

### Patients

Patients with thyroid nodules who underwent FNAC detection and BRAF^V600E^ mutation detection at the China-Japan Union Hospital of Jilin University from July 2021 to December 2021 were selected for this study. Patients who eventually underwent surgery as well as had a complete cytological diagnosis, BRAF^V600E^ mutation detection and postoperative pathological results were included in the study. The diagnostic criteria for HT are as follows: (1) thyroid enlargement, tendiness, large or asymmetric isthmus, or with nodules; (2) Patients with typical clinical manifestations, as long as the blood TgAb or TPOAb positive, can be diagnosed; (3) For those with atypical performance, a high titer of anti-thyroid antibody is required for diagnosis, that is, when two antibodies are measured by radioimmunoassay, the results of two consecutive tests are greater than or equal to 60%; (4) In patients with hyperthyroidism at the same time, the above high titer antibody persisted for more than half a year; (5) Thyroid puncture biopsy has diagnostic value, and ultrasound examination has certain significance in the diagnosis of this disease.

The inclusion criteria were as follows: (1) Ultrasound manifestations of thyroid nodules are indicative of malignancy i.e., the nodules are irregular in shape, ill-defined and hypoechoic, with an aspect ratio greater than 1. Further, punctate calcifications can be seen;(2)the patient underwent FNAC examination and BRAF^V600E^ mutation detection at the same time;(3)the patient received surgical treatment and had a complete cytological diagnosis, BRAF^V600E^ mutation detection and postoperative pathological results. The exclusion criteria were as follows:(1)patients with malignant tumors of other organs in addition to the thyroid,(2)Postoperative pathology included thyroid malignancies other than PTC,(3)patients who underwent thyroid reoperation;(4)incomplete clinical data.

### Puncture method

The patient was placed in a supine position. Next, a sterile drape was placed in front of the neck and the skin was disinfected with 0.5% iodophor. Thereafter, local infiltration anesthesia was performed with 2% lidocaine. Under the guidance of ultrasound, the surgeon applied a 22-gauge disposable puncture biopsy needle into the center of the nodule and repeatedly aspirated (2-3 times) at multiple points. For the preparation of pathological sections, 3-4 smears were obtained from the puncture and fixed with 95% ethanol. The remaining puncture was placed in an EP tube for BRAF^V600E^ mutation detection.

### Cytological diagnostic methods

All FNAC specimens were diagnosed by a cytopathologist and classified into the following 6 categories according to the Bethesda System for Reporting Thyroid Cytopathology: category I, nondiagnostic or unsatisfactory biopsy; category II, benign (i.e., nodular goiter, colloid goiter, hyperplastic/adenomatoid nodule, Hashimoto’s thyroiditis); category III, atypia of undetermined significance (AUS) or follicular lesion of undetermined significance (FLUS); category IV, follicular neoplasm or suspicious for a follicular neoplasm (FN/SFN); category V, suspicious for malignancy; and category VI, malignancy. In this study, categories I-IV were classified as cytologically negative and categories V-VI were classified as cytologically positive.

### BRAF^V600E^ mutation detection

DNA was extracted from FNA specimens using the QIAamp DNA FFPE Tissue Kit, and the DNA concentration was measured by a spectrophotometer. The detection was performed using the QX200 Droplet Digital PCR System (Bio-Rad, USA). The reaction conditions for PCR were as follows:95°C for 10 min, 94°C for 30 s, 55°C for 60 s, the total number of cycles: 40, 98°C 10 min, finished at 4°C. The test results were judged as positive if ≥3 points fall in the “ch1+ ch2-” area.

The primer sequences were as follows: Forward primer: 5’-CTACTGTTTTCCTTTACTTACTACACCTCAGA-3’; reverse primer: 5’-ATCCAGACAACTGTTCAAACTGATG-3’.

FNAC and BRAF^V600E^ mutations tests were positive. Nodules classified by Bethesda as V-VI or BRAF^V600E^ mutation-positive or both were classified as positive.

### Statistical analysis

All statistical analysis was done by the SPSS (version: 25) software. Taking postoperative pathology as the gold standard, the diagnostic efficacy of FNAC and FNAC combined with BRAF^V600E^ mutation detection on PTC with HT was compared. The count data were expressed as a rate (%). Chi-square analysis and Fisher’s exact test were used to compare categorical variables. P<no><0.05</no> was considered statistically significant.

## Results

### Patient characteristics

A total of 122 patients were included, including 111 patients with postoperative, pathologically confirmed PTC and 11 patients with nodular goiter. Among them, 102 (83.6%) were female and 20 (16.4%) were male. The mean age of the patients was 44.17 ± 9.11 years. Among them, 56 patients were aged ≥55 years old and accounted for approximately 45.9% of all the patients. Further, 66 patients (54.1%) were <55 years old. Preoperative FNA results were positive in 105 patients (86.1%). A total of 60 (49.2%) patients carried the BRAF^V600E^ mutation. (**Table 1**).

**Table 1:** Basic characteristics of patients.

Clinical features	Total(%)
Sex	
Female	102(83.6)
Male	20(16.4)
Age	
Average	44.17 ± 9.11
<55	66(54.1)
≧55	56(45.9)
FNA	
Negative	17(13.9)
Positive	105(86.1)
BRAF^V600E^	
Wild type	62(50.8)
Mutant	60(49.2)

### Preoperative FNAC results and BRAF^V600E^ mutation in patients with thyroid nodules with HT background

Preoperative FNAC results of patients with thyroid nodules with HT demonstrated that there were 2 benign patients, and none of them were associated with BRAF^V600E^ mutation. There were 15 cases of AUS/FLUS, of which 4 cases carried the BRAF^V600E^ mutations. Further, there were 7 suspected malignant cases and 3 of them had BRAF^V600E^ mutation. Of the 98 malignant cases, 53 had BRAF^V600E^ mutation (**Table 2**).

**Table 2:** Preoperative FNAC results and BRAFV600E mutation in thyroid nodules patients with HT.

FNAC	Count	BRAF^V600E^	
		Mutant	Wild type
I	0	0	0
II	2	0	2
III	15	4	11
IV	0	0	0
V	7	3	4
VI	98	53	45

### Diagnostic value of FNAC and FNAC combined with BRAF^V600E^ mutation detection in thyroid nodules patients with HT

A total of 122 patients who had thyroid nodules with HT underwent surgery. Of these, 111 patients were confirmed with PTC by postoperative pathology and 11 patients had a nodular goiter. Preoperative FNAC results were positive in 105 patients—of these, 104 patients were diagnosed with PTC postoperatively. Furthermore, 17 patients had negative FNAC results and 7 of them were diagnosed with PTC after surgery (**Table 3**). All 60 patients who had positive BRAF^V600E^ mutation before surgery were diagnosed with PTC by paraffin pathology. Further, 62 patients had negative BRAF^V600E^ mutation before surgery. Among these, 51 patients were diagnosed with PTC by paraffin pathology (**Table 4**). A total of 109 patients tested positive for FNAC combined with BRAF^V600E^ mutation before surgery, of which 108 patients were diagnosed with PTC by postoperative pathology. Further, 13 patients were negative for FNAC combined with BRAF^V600E^ mutation detection; of these, 3 patients were diagnosed with PTC by postoperative pathology (**Table 5**). The sensitivity of preoperative FNAC in the diagnosis of PTC with HT was 93.69%, the specificity was 90.90% and the accuracy was 93.44%. The sensitivity, specificity and accuracy of FNAC combined with BRAF^V600E^ mutation detection were 97.30%, 90.90% and 96.72%, respectively. The sensitivity, as well as the accuracy of FNAC in combination with BRAF^V600E^ mutation detection, was higher as compared to FNAC alone, and the difference was statistically significant (p < 0.05) (**Table 6**). FNAC combined with BRAF^V600E^ mutation detection can improve the sensitivity and accuracy of the diagnosis of benign and malignant thyroid nodules in the background of HT.

**Table 3:** FNAC and postoperative pathological results.

FNAC	Histological pathology		Total
	Malignant	Benign	
Positive	104(99.0%)	1(1.0%)	105
negative	7(41.2%)	10(58.8%)	17
Total	111	11	122

**Table 4:** BRAF^V600E^ mutations and postoperative pathological results.

BRAF^V600E^	Histological pathology		Total
	Malignant	Benign	
Mutant	60(100.0%)	0(0.0%)	60
Wild type	51(82.3%)	11(17.7%)	62
Total	111	11	122

**Table 5:** FNAC combined with BRAF^V600E^ mutation detection and postoperative pathological results.

FNAC combined with BRAF^V600E^	Histological pathology		Total
	Malignant	Benign	
Positive	108(99.1%)	1(0.9%)	109
Negative	3(23.1%)	10(76.9%)	13
Total	111	11	122

**Table 6:** Diagnostic efficacy of FNAC alone versus combination testing.

	Sensitivity(%)	Specificity(%)	Accuracy(%)
FNAC	104/111(93.69)	10/11(90.90)	114/122(93.44)
Combination detection	108/111(97.30)	10/11(90.90)	118/122(96.72)
*p*	<0.001	0.091	<0.001

## Discussion

The presence of Hashimoto’s thyroiditis increases the difficulty of diagnosing benign and malignant thyroid nodules. HT ultrasound showed that diffuse hypoechoic, glandular parenchyma echogenic disorder and pseudo-nodules were also observed. This may affect the accurate identification of thyroid nodules during FNAC. Previous studies have shown that thyroiditis is one of the most common factors in false-positive PTC diagnoses (12). The strong overlap of morphological features in HT and PTC can present challenges for cytopathologists—experienced cytopathologists may also be uncertain about the diagnosis of the two diseases. Moreover, diagnostic pitfalls in the assessment of HT cytology may vary by disease stage. During the “cellular stage,” a large number of eosinophils proliferate. Such changes in eosinophils may lead to nuclear atypia i.e., enlarged nucleus, finely textured chromatin, prominent nuclear membrane and large nucleoli. Occasional nuclear grooves or pseudo inclusions may lead to the overdiagnosis of PTC. In contrast, in the “fibrotic stage”, the thyroid tissue is extensively fibrotic and sclerotic, and few cells may be extracted at the time of FNAC. In this case, the presence of some atypical cells is suggestive of PTC. Further, the insufficient number of cells may affect the accuracy of the diagnosis, consequently the number of Bethesda class III and IV increased (13).

BRAF^V600E^ mutation is a common mutation in PTC that leads to abnormal activation of the MAPK pathway, which in turn plays a key role in the occurrence and development of PTC (14–16). Since BRAF^V600E^ mutation rarely occurs in benign thyroid lesions, its diagnostic specificity is high. The American Thyroid Association recommends the detection of relevant molecular markers for nodules whose benign and malignant nodules cannot be determined by FNAC (17). It has been reported that FNAC combined with preoperative BRAF^V600E^ mutation detection can significantly improve the diagnostic performance of thyroid nodules as compared to FNAC alone (18). However, there are a few studies on the effect of FNAC combined with BRAF^V600E^ mutation detection on the diagnostic performance of PTC in the context of HT.

In our study, a total of 122 patients who had thyroid nodules with HT underwent FNAC and BRAF^V600E^ mutation testing as well as surgery. Postoperative pathology confirmed the diagnosis of PTC in 111 patients and nodular goiter in 11 patients. We did not detect BRAF^V600E^ mutation in all the benign lesions. Further, 60 patients for whom BRAF^V600E^ mutation was detected preoperatively were diagnosed with PTC by postoperative pathology. Notably, BRAF^V600E^ mutation detection has high specificity for the diagnosis of PTC with HT. The results showed that preoperative FNAC combined with BRAF^V600E^ mutation detection could improve the sensitivity of FNAC in diagnosing PTC in the background of HT from 93.69% to 97.30% and accuracy from 93.44% to 96.72%. The increase in sensitivity was mainly from patients with FNAC results of AUS/FLUS. In this study, 15 patients with HT had FNAC results of AUS/FLUS and 4 of them carried the BRAF^V600E^ mutation. PTC was confirmed for these patients by postoperative pathology. It was noted that the combination of BRAF^V600E^ mutation detection before surgery could help in improving the accuracy of diagnosis in such patients and avoiding lapses in determining malignant cases. The sensitivity and accuracy of the diagnosis of FNAC in our study were high. This may be because the surgeons who performed preoperative ultrasound assessment and puncture, and the pathologists who performed the cytological diagnosis were all senior physicians with rich clinical experience. Even with the interference of Hashimoto’s thyroiditis background, they were able to select suspected nodules for FNAC detection more accurately than junior physicians. But the combined testing can still improve the sensitivity and accuracy of diagnosis, so we speculate that the combined testing is more helpful for less experienced primary physicians. In addition, the number of patients diagnosed with benign lesions after surgery was fewer in this study. Surgeon combined BRAF V600E mutation testing, cytological diagnosis, and ultrasound to select only patients with high risk of malignancy for surgery. Due to the small number of patients with negative FNAC results but BRAF^V600E^ mutation positivity and eventual surgical treatment, the specificity of the combined testing in this study was the same as that of FANC, and we were unable to accurately assess the effect of the combined test on the specificity of the preoperative diagnosis. Our results show that preoperative FNAC combined with BRAF^V600E^ mutation detection can help in improving the diagnostic efficacy of PTC in the context of HT to some extent, and this may be of greater help to less experienced primary physicians.

However, this study had certain limitations. First, most of the patients who received surgical treatment in this study were patients with preoperative Bethesda categories V and VI. In addition, the number of patients with thyroid follicular degeneration is relatively small, and our sample size is limited, resulting in no Bethesda categories IV patients in the study. For Bethesda categories III patients, surgery is performed only in patients who are highly suspected of malignancy on ultrasound and carry BRAF V600E mutation, or who are negative for BRAF V600E mutation but are anxious, refuse close follow-up, or repeat puncture. Some patients with negative cytology and BRAF^V600E^ mutation were unable to undergo surgical treatment. This had an impact on the diagnostic performance of PTC in the context of HT. Second, fine-needle aspiration cytology was performed by the same senior doctor at our institution. Although there was reliability and comparability in this approach, the impact of FNAC and combined testing on the diagnostic performance of inexperienced doctors has not been considered in this study. Long-term follow-up studies with larger patient samples from multiple institutions are needed to further determine the diagnostic value of the combination test.

## Data availability statement

The raw data supporting the conclusions of this article will be made available by the authors, without undue reservation.

## Ethics statement

All experimental procedures involving humans in this study were reviewed and approved by the Ethics Committee of the China-Japan Union Hospital of Jilin University. Written informed consent was obtained from all patients.

## Author contributions

JF: Formal analysis, Writing – original draft, Data curation, Validation. XY: Conceptualization, Writing – original draft. XCW: Formal analysis, Writing – original draft. SX: Software, Writing – original draft. XJW: Data curation, Writing – original draft. CD: Investigation, Writing – original draft. WY: Methodology, Writing – original draft. GZ: Funding acquisition, Writing – review & editing.

## References

[1] Xie Z Lun Y Li X He Y Wu S Wang S. Bioinformatics analysis of the clinical value and potential mechanisms of AHNAK2 in papillary thyroid carcinoma. Aging (Albany NY). (2020) 12:18163–80. doi: 10.18632/aging.v12i18 PMC7585101 32966238

[2] Nikiforov YE. Role of molecular markers in thyroid nodule management: then and now. Endocr Pract. (2017) 23:979–88. doi: 10.4158/EP171805.RA 28534687

[3] Yi KI Ahn S Park DY Lee JC Lee BJ Wang SG. False-positive cytopathology results for papillary thyroid carcinoma: A trap for thyroid surgeons. Clin Otolaryngol. (2017) 42:1153–60. doi: 10.1111/coa.12840 28130940

[4] Gao L Ma B Zhou L Wang Y Yang S Qu N. The impact of presence of Hashimoto’s thyroiditis on diagnostic accuracy of ultrasound-guided fine-needle aspiration biopsy in subcentimeter thyroid nodules: A retrospective study from FUSCC. Cancer Med. (2017) 6:1014–22. doi: 10.1002/cam4.997 PMC5430084 28382784

[5] National Health Commission of the People’s Republic of China. Thyroid Cancer Diagnosis and Treatment Standards (2018 Edition). Chinese General Surgery Literature. (2019) 13.01:1–15. doi: 10.3877/cma.j.issn.1674-0793.2019.01.001

[6] Lu HZ Zhang N Liu W Zhu XY Qi D Wang Y. [Differential protein expressions in papillary thyroid carcinoma patients with or without Hashimoto’s thyroiditis]. Zhonghua Zhong Liu Za Zhi. (2020) 42:463–8. doi: 10.3760/cma.j.cn112152-20191219-00824 32575941

[7] Rhoden KJ Unger K Salvatore G Yilmaz Y Vovk V Chiappetta G. RET/papillary thyroid cancer rearrangement in nonneoplastic thyrocytes: follicular cells of Hashimoto’s thyroiditis share low-level recombination events with a subset of papillary carcinoma. J Clin Endocrinol Metab. (2006) 91:2414–23. doi: 10.1210/jc.2006-0240 16595592

[8] Guo H-q Zhao H Zhang Z-h Zhu Y-l Xiao T Pan Q-j. Impact of molecular testing in the diagnosis of thyroid fine needle aspiration cytology: data from mainland China. Dis Markers. (2014) 2014:912182. doi: 10.1155/2014/912182 24591770 PMC3925579

[9] Jeong D Jeong Y Park JH Han SW Kim SY Kim YJ. BRAFV600E mutation analysis in papillary thyroid carcinomas by peptide nucleic acid clamp real-time PCR. Ann Surg Oncol. (2013) 20:759–66. doi: 10.1245/s10434-012-2494-0 23179992

[10] Marina N Nikiforova 1 Kimura ET Gandhi M Biddinger PW Knauf JA Basolo F. BRAF mutations in thyroid tumors are restricted to papillary carcinomas and anaplastic or poorly differentiated carcinomas arising from papillary carcinomas. J Clin Endocrinol Metab. (2003) 88:5399–404. doi: 10.1210/jc.2003-030838 14602780

[11] Al-Salam S Sharma C Afandi B Al Dahmani K Al-Zahrani AS Al Shamsi A. BRAF and KRAS mutations in papillary thyroid carcinoma in the United Arab Emirates. PloS One. (2020) 15:e0231341–e. doi: 10.1371/journal.pone.0231341 PMC7173769 32315324

[12] Baloch ZW LiVolsi VA. Current role and value of fine-needle aspiration in nodular goitre. Best Pract Res Clin Endocrinol Metab. (2014) 28:531–44. doi: 10.1016/j.beem.2014.01.010 25047204

[13] Zhu Y Song Y Xu G Fan Z Ren W. Causes of misdiagnoses by thyroid fine-needle aspiration cytology (FNAC): our experience and a systematic review. Diagn Pathol. (2020) 15:1. doi: 10.1186/s13000-019-0924-z 31900180 PMC6942345

[14] Kim TH Park YJ Lim JA Ahn HY Lee EK Lee YJ. The association of the BRAFV600E mutation with prognostic factors and poor clinical outcome in papillary thyroid cancer. Cancer. (2012) 118:1764–73. doi: 10.1002/cncr.26500 21882184

[15] Sezer H Uren N Yazici D. Association between BRAF(V600E) mutation and the clinicopathological features in incidental papillary thyroid microcarcinoma: A single-center study in Turkish patients. North Clin Istanb. (2020) 7:321–8. doi: 10.14744/nci.2020.69586 PMC7521097 33043255

[16] Choi S Yi Park H Kang MK Lee DK Lee KD Lee HS. The relationship between the BRAF(V600E) mutation in papillary thyroid microcarcinoma and clinicopathologic factors. World J Surg Oncol. (2013) 11:291–. doi: 10.1186/1477-7819-11-291 PMC3833185 24228637

[17] Haugen BR. 2015 American Thyroid Association Management Guidelines for Adult Patients with Thyroid Nodules and Differentiated Thyroid Cancer: What is new and what has changed? Cancer. (2017) 123:372–81. doi: 10.1002/cncr.30360 27741354

[18] Qi W Shi C Zhang P Feng L Wang J Chen D. Effect of BRAF V600E mutation detection of fine-needle aspiration biopsy on diagnosis and treatment guidance of papillary thyroid carcinoma. Pathol Res Pract. (2020) 216:153037. doi: 10.1016/j.prp.2020.153037 32703500

